# Novel Hybrid Adaptive Controller for Manipulation in Complex Perturbation Environments

**DOI:** 10.1371/journal.pone.0129281

**Published:** 2015-06-01

**Authors:** Alex M. C. Smith, Chenguang Yang, Hongbin Ma, Phil Culverhouse, Angelo Cangelosi, Etienne Burdet

**Affiliations:** 1 Centre for Robotics and Neural Systems, Plymouth University, Plymouth, UK; 2 College of Automation Science and Engineering, South China University of Technology, Guangzhou, China; 3 State Key Laboratory of Intelligent Control and Decision of Complex Systems, Beijing Institute of Technology, Beijing, China; 4 School of Automation, Beijing Institute of Technology, Beijing, China; 5 Department of Bioengineering, Imperial College London, London, UK; University of Vermont, UNITED STATES

## Abstract

In this paper we present a hybrid control scheme, combining the advantages of task-space and joint-space control. The controller is based on a human-like adaptive design, which minimises both control effort and tracking error. Our novel hybrid adaptive controller has been tested in extensive simulations, in a scenario where a Baxter robot manipulator is affected by external disturbances in the form of interaction with the environment and tool-like end-effector perturbations. The results demonstrated improved performance in the hybrid controller over both of its component parts. In addition, we introduce a novel method for online adaptation of learning parameters, using the fuzzy control formalism to utilise expert knowledge from the experimenter. This mechanism of meta-learning induces further improvement in performance and avoids the need for tuning through trial testing.

## Introduction

Modern robots are expected to interact extensively with the environment and with humans [1, 2]. This interaction with dynamic and unknown environments requires a control method that maintains stability and task effectiveness despite disturbances. One of the first schemes proposed to control interaction with an unknown environment is impedance control [3]. The environment is modeled as an admittance and the manipulator as an impedance, so that interactive control is achieved through the exchange of energy. Impedance control can be designed on top of adaptive control, which compensates parametric uncertainties [4–6]. Adaptive impedance control methods, developed in [7–9], have improved the operational performance of a traditional impedance controller. In particular, the work in [9] shows how stability and successful performance can be gradually acquired despite the initial interaction instability typical of tool use such as drilling or carving [10].

**Fig 1:**
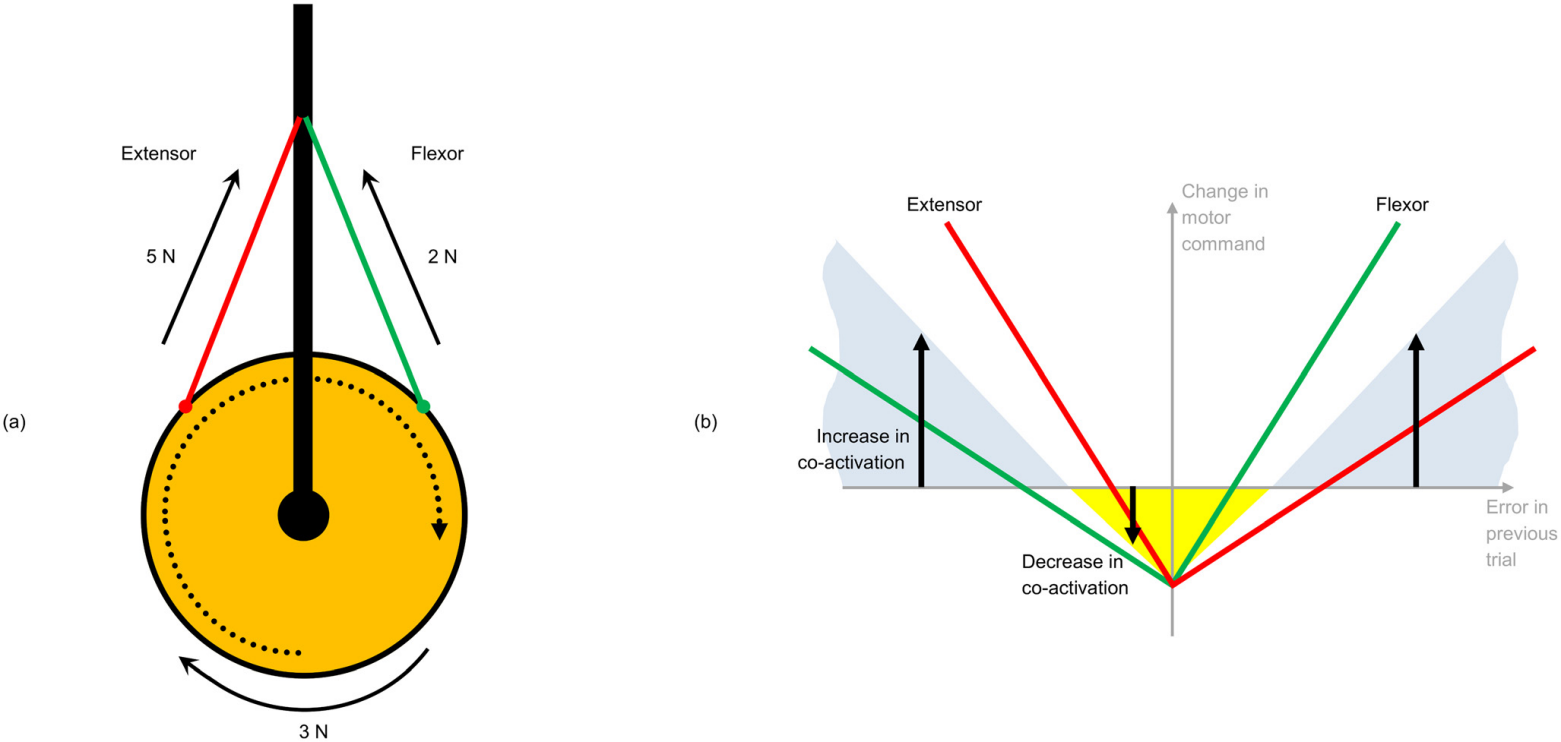
How co-contraction affects muscle impedance. (a): By contracting at the same time with different forces, the flexor and extensor muscles work together to maintain effector torque, but with increased impedance. (b): the “v-shape” of the adaptive law. Impedance increases irrespective of error direction, and decreases when error is below a threshold; this mechanism ensures minimisation of metabolic cost (i.e. control effort).

Parallel to these developments, studies have shown that the human nervous system can adapt mechanical impedance (e.g. the resistance to perturbations) to succeed in performing tasks in stable and unstable environments [11, 12]. This is achieved through co-contraction of agonist/antagonist muscle groups, as demonstrated in Fig 1(a). The nervous system adapts motor commands to stabilise interactions through independent control of impedance and exerted force; the adaptation automatically selects suitable muscle activations to compensate for the interaction force and instability. At the same time, metabolic cost is minimised through the natural relaxation of muscle groups when error is sufficiently small. A model for this learning was introduced in [13, 14], which gave rise to a novel kind of non-linear adaptive controller that has been successfully demonstrated on robots [15]. The adaptation of impedance in this biomimetic controller follows a “v-shaped” algorithm, as shown in Fig 1(b). Conventionally designed adaptive control designs are typically focussed on the estimation of uncertain parameters under stable motion [16]; in comparison, the biomimetic control design is able to acquire stability in unstable dynamics as well as minimise control effort, through adaptation of force and impedance [9]. Similar to muscle relaxation, under stable interaction the controller also demonstrates compliance, which has received much attention in recent research on robotic manipulation [17] [18].

The present paper extends this novel adaptive controller in two aspects: the first contribution is *hybrid task-space/joint-space control*. Controllers are typically implemented in either joint space (corresponding to the actuators) or in Cartesian space (in which case the inverse kinematics must be solved). Both of these control methods have advantages and disadvantages:

- In contrast to joint space controllers, Cartesian controllers allows for intuitive trajectories in the world space. Objects placed in the workspace typically have a Cartesian representation, e.g. a box placed 0.1 metres in front of the robot.
- On the other hand, robots typically require inputs in joint-space, i.e. torques rather than forces and moments. Therefore, joint space control is less computationally expensive than Cartesian space control, as it avoids the inverse kinematic problem. This is especially true for under-actuated or redundant robots like the Baxter manipulator.
- Telepresence tasks may be more intuitive in joint space, when an anthropomorphic robot is imitating a human operator.

More specifically to this work,

- Joint control can make the manipulator robust against disturbances along any part of the arm by monitoring joint-space errors.
- Cartesian control is sensitive to task-specific disturbances occurring at the end-effector.

Therefore, a *hybrid joint-Cartesian space control scheme* is developed and investigated in this paper to take advantages of these two control approaches. The Cartesian task we study is that of carrying an object along a given trajectory while disturbances are applied either on the endpoint or along the arm (or both), similar to noise rejection when holding a glass of champagne in a crowded room [19]. This extends developments found in [20] and [21].

Another aspect of adaptive control that has received little attention is *the setting of learning parameters*. These parameters are typically tuned by the user, in order to complete the task and improve performance, e.g. by minimising the tracking error. Automating the selection of learning parameters is not an easy task. Real-world manipulator systems have complex and unknown dynamics due to interaction with the environment, which is difficult—or in some cases, impossible—to model. The neural network-based approach of [22, 23] may be used to estimate uncertainties in order to avoid some of these problems. However, fuzzy logic can be used to transfer expertise from a human operator in order to make rational decisions in the face of imprecise data [24–26]. Fuzzy logic has been successfully introduced into control systems to improve performance [27], and recently has been used in non-linear control systems [28] and robot manipulation [29]. This paper thus develops a method based on fuzzy logic to set the learning parameters.

**Fig 2:**
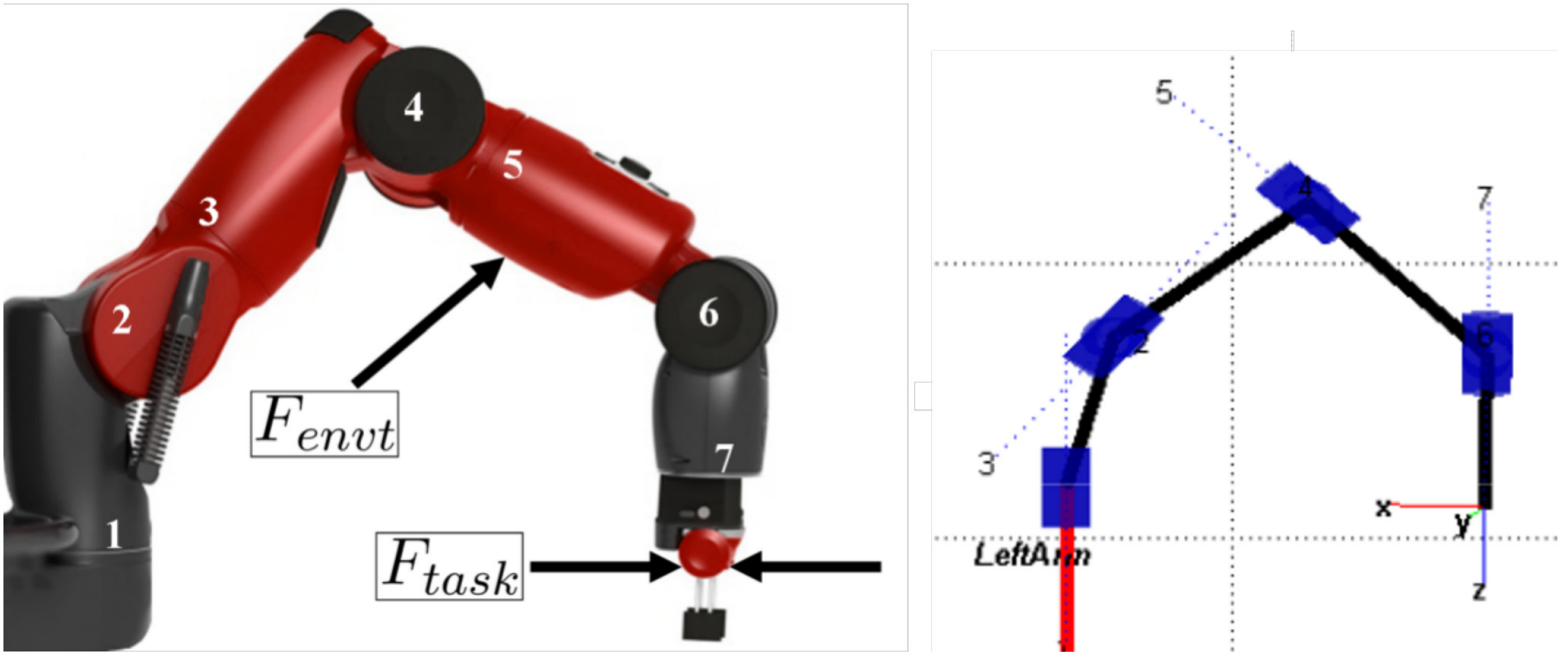
Baxter arm, and disturbance forces acting on it. *F*_*task*_ acts at the end-effector and *F*_*envt*_ is applied further up the arm as described in Eqs (2) and (4). The model generated using MATLAB and Peter Corke’s Robotics Toolbox is shown on the right [37].

The concepts of this paper will be simulated and tested on one arm of the Baxter robot (Fig 2). Baxter is a bimanual, low cost robot, designed for introductory industrial applications from Rethink Robotics©, which has recently become available in a research version for use in academia.

## Control problem

Baxter is required to move along a given trajectory under the influence of a high frequency, low amplitude vibration at the end-effector, simulating the type of disturbance a tool might produce. In addition, a high amplitude and low frequency perturbation is applied to a point on the arm away from the end-effector, to simulate collision with an operator or with the environment. For reference, nomenclature is provided in Table 1.

**Table 1:** Nomenclature.

Symbol	Description
*n*	Number of joints, i.e. degrees of freedom
*q* ∈ ℜ^*n*^	Joint angle
$\dot{q}\in {ℜ}^{n}$	Joint angular velocity
$\overset{..}{q}\in {ℜ}^{n}$	Joint angular acceleration
*X* ∈ ℜ^6^	Cartesian/task-space position
$\dot{X}\in {ℜ}^{6}$	Cartesian/task-space velocity
$\overset{..}{X}\in {ℜ}^{6}$	Cartesian/task-space acceleration
q*, X*	Desired joint position, Cartesian position
*F*_*task*_, *F*_*envt*_ ∈ ℜ^6^	Internal, external force respectively
*M* ∈ ℜ^*n*×*n*^	Inertia matrix
*C* ∈ ℜ^*n*^	Coriolis and Centrifugal force
*G* ∈ ℜ^*n*^	Force due to gravity
*τ*_*u*_ ∈ ℜ^*n*^	Input torque
*τ*_*dist*_ ∈ ℜ^*n*^	Torque due to disturbances
*τ*_*r*_ ∈ ℜ^*n*^	Reference torque
*τ*_*j*_ ∈ ℜ^*n*^	Joint-space control torque
*τ*_*x*_ ∈ ℜ^*n*^	Task-space control torque
*L* ∈ ℜ^*n*×*n*^	Stability margin
*J* ∈ ℜ^6×*n*^	Manipulator Jacobian matrix
*J* ^†^ ∈ ℜ^*n*×6^	Pseudo-inverse Jacobian
*Z* ∈ ℜ^6×6^	Reduction matrix
*e*, *e*_*x*_	Joint, task-space position error, respectively
$\dot{e},{\dot{e}}_{x}$	Joint, task-space velocity error
*ɛ*_*j*_, *ɛ*_*x*_	Joint, task-space tracking error
|⋅|	Absolute value
‖⋅‖	Euclidian vector norm
0_[*i* × *j*]_	*i* × *j*—dimensional zero matrix

### Robot Dynamics

The robot arm dynamics are given as:

$$\begin{array}{c} M\left(q\right)\ddot{q}+C\left(q,\dot{q}\right)\dot{q}+G\left(q\right)=\tau_{u}+\tau_{dist} \end{array}$$ (1)

where *q* denotes the vector of joint angles, *M*(*q*) ∈ ℝ^*n*×*n*^ is the symmetric, bounded, positive definite inertia matrix, and *n* is the degree of freedom (DoF) of the robot arm; $C(q,\dot{q})\dot{q}\in {\mathbb{R}}^{n}$ denotes the Coriolis and Centrifugal force; *G*(*q*) ∈ ℝ^*n*^ is the gravitational force; *τ*_*u*_ ∈ ℝ^*n*^ is the vector of control input torque; and *τ*_*dist*_ ∈ ℝ^*n*^ is the disturbance torque caused by friction, environmental disturbances or loads as described in the next section. The control torques *τ*_*u*_ are generated by the designed controllers in order to achieve desired performance in terms of motion tracking and disturbance rejection.

### Disturbances

We assume that the disturbance torque *τ*_*dist*_ can be broken down to two components to simulate both a task disturbance at the end effector, described here as *F*_*task*_, and an environmental disturbance *F*_*envt*_ applied on the arm, as shown in Fig 2:

$$\begin{array}{c} F_{task}\equiv {\left[p\;0\;0\;0\;0\;0\right]}^{T},\;p\equiv A_{p}\sin\left(2\pi \omega_{p}t\right) \end{array}$$ (2)

is applied on the endpoint, where 0 < *A*_*p*_ ≤ 20 is the amplitude and 100 < *ω*_*p*_ ≤ 1000 the frequency of oscillation in Hertz. In joint space, the torque applied is then

$$\begin{array}{c} \tau_{task}=J^{T}\left(q\right)\;F_{task} \end{array}$$ (3)

where the Jacobian *J*(*q*) is defined through $\dot{x}\equiv J(q)\dot{q}$. The environmental disturbance is given by

$$\begin{array}{c} F_{envt}\equiv {\left[r\;0\;0\;0\;0\;0\right]}^{T},\;r=A_{r}\sin\left(2\pi \omega_{r}t\right) \end{array}$$ (4)

where 20*N* < *A*_*r*_ ≤ 100*N* is the perturbation amplitude, similar to average limits of human push/pull strength [30], and 0.1 < *ω*_*r*_ ≤ 1 the frequency in Hertz, which provides a slowly changing disturbance. To simulate the environmental force *F*_*envt*_ being applied at a point on the arm, e.g. at the elbow, the Jacobian matrix *J* is reduced by a matrix *Z*, defined as

$$\begin{array}{c} Z\equiv [\begin{array}{c} I_{\left[z\times z\right]}\\ 0_{\left[(n-z)\times z\right]} \end{array}] \end{array}$$ (5)

where *z* is the number of joints from the base to the contact point; e.g. if the force is applied on the elbow, *z* = 4. The torque can then be derived as

$$\begin{array}{c} \tau_{envt}={\left(J(q)\;Z\right)}^{\;T}\;F_{envt} \end{array}$$ (6)

The disturbance torque *τ*_*dist*_ in Eq (1) is comprised of a combination of terms in Eqs (6) and (3).

## Adaptive Control

### Feedforward controller

Given the dynamics of a manipulator in Eq (1), we employ the following controller as the initial torque input

$$\begin{array}{c} \tau_{r}\left(t\right)=M\left(q\right){\ddot{q}}^{*}+C\left(q,\dot{q}\right){\dot{q}}^{*}+G\left(q\right)-L\left(t\right)\varepsilon \left(t\right)\;. \end{array}$$ (7)

where *L*(*t*)*ɛ*(*t*) corresponds to a desired *stability margin* [9] which produces minimal feedback (similar to the passive impedance effect of muscles and tendons), and the first three terms are feed-forward compensation for the manipulator’s dynamics. As in sliding mode control, we use the *tracking error*

$$\begin{array}{c} \varepsilon \left(t\right)=\dot{e}\left(t\right)+\kappa e\left(t\right) \end{array}$$ (8)

where

$$\begin{array}{c} e\left(t\right)=q\left(t\right)-q^{*}\left(t\right),\;\dot{e}\left(t\right)=\dot{q}\left(t\right)-{\dot{q}}^{*}\left(t\right) \end{array}$$ (9)

are joint angle and angular velocity errors, respectively. In addition to the above control input *τ*_*r*_(*t*), we develop two adaptive controllers in joint space and task space as follows.

#### Joint space adaptive control

The human-like adaptive law for tuning the feed-forward and feedback components of the control torque *τ*_*u*_ from [9] is applied both in joint and task spaces. The adaptation here is continuous during movement, rather than trial after trial on repeated movements, so that tracking error and effort are continuously minimised. Let us define

$$\begin{array}{c} \tau_{j}\left(t\right)=-\tau \left(t\right)-K\left(t\right)e\left(t\right)-D\left(t\right)\dot{e}\left(t\right) \end{array}$$ (10)

where −*τ*(*t*) is the learned *feed-forward* torque, and −*K*(*t*)*e*(*t*) and $-D(t)\dot{e}(t)$ are *feedback* torque terms due to stiffness and damping, respectively. The adaptive laws introduced in [9] for a trajectory of period *T* are given as:

$$\begin{array}{ccc} \delta \tau (t) & \equiv & \tau \left(t\right)-\tau \left(t-T\right)\equiv Q_{\tau }(\varepsilon (t)-\gamma (t)\tau (t)),\\ \delta K(t) & \equiv & K\left(t\right)-K\left(t-T\right)\equiv Q_{K}(\varepsilon \left(t\right)e^{T}\left(t\right)-\gamma \left(t\right)K\left(t\right)),\\ \delta D(t) & \equiv & D\left(t\right)-D\left(t-T\right)\equiv Q_{D}(\varepsilon \left(t\right){\dot{e}}^{T}\left(t\right)-\gamma \left(t\right)D\left(t\right))\;. \end{array}$$ (11)

**Fig 3:**
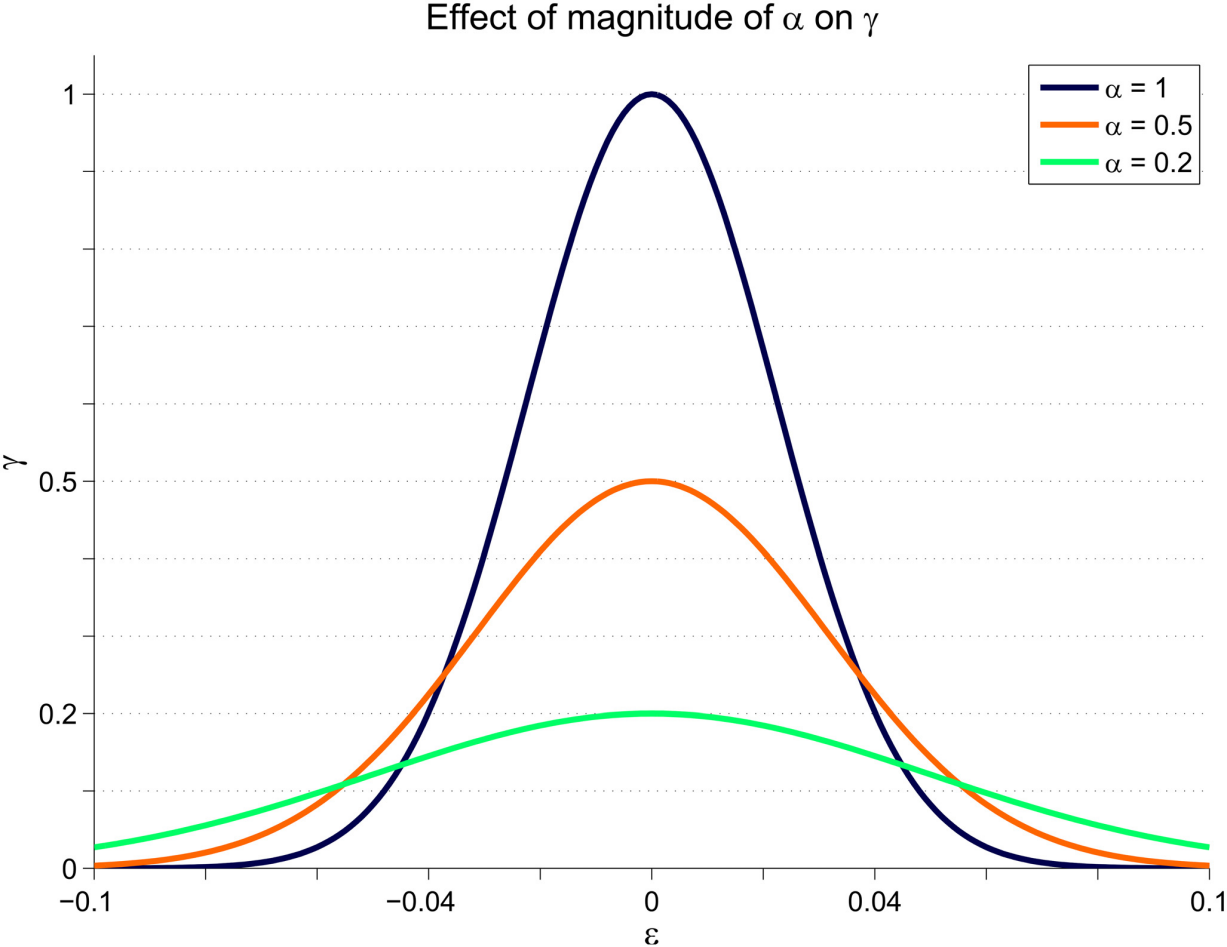
How the magnitude of *α* affects the forgetting factor *γ*. Higher values of *α* have a high narrow shape, so that when tracking performance is good the control effort is reduced maximally. When tracking performance is poor, the forgetting factor is small, increasing applied feedback torque.

In the present paper we decouple the forgetting factor *γ*(*t*) from the gain matrices *Q*_(⋅)_ in order to avoid high frequency oscillation, which can occur when both *γ* and *Q*_(⋅)_ are large. As mentioned above, we consider the adaptation in continuous time, rather than by iteration over consecutive trials, yielding the *joint space adaptation laws*:

$$\begin{array}{ccc} \delta \tau (t) & \equiv & \tau \left(t\right)-\tau \left(t-\delta t\right)\equiv Q_{\tau }\;\varepsilon_{j}\left(t\right)-\gamma_{j}\left(t\right)\;\tau \left(t\right)\;,\\ \delta K_{j}\left(t\right) & \equiv & K_{j}\left(t\right)-K_{j}\left(t-\delta t\right)\equiv Q_{Kj}\;\varepsilon_{j}\left(t\right)e_{j}^{T}\left(t\right)-\gamma_{j}\left(t\right)\;K_{j}\left(t\right)\;,\\ \delta D_{j}\left(t\right) & \equiv & D_{j}\left(t\right)-D_{j}\left(t-\delta t\right)\equiv Q_{Dj}\;\varepsilon_{j}\left(t\right){\dot{e}}_{j}^{T}\left(t\right)-\gamma_{j}\left(t\right)\;D_{j}\left(t\right) \end{array}$$ (12)

where *δt* is the sampling time, *K*_*j*_(0) = 0_[*n*×*n*]_ and *D*_*j*_(0) = 0_[*n*×*n*]_. *Q*_*τ*_, *Q*_*Kj*_, *Q*_*Dj*_ ∈ ℜ^*n*×*n*^ are diagonal positive-definite gain matrices. Furthermore, in [9], *γ*(*t*) ∈ ℜ^*n*×*n*^ was diagonal with

$$\begin{array}{c} \gamma_{ii}\left(t\right)=\frac{a}{1+b{∥\varepsilon_{i}\left(t\right)∥}^{2}} \end{array}$$ (13)

which requires two tuning variables, *a* and *b*. To simplify parameter selection, *γ* is redefined as

$$\begin{array}{c} \gamma_{ii}\left(t\right)=\alpha_{j_{i}}\;\exp(-\frac{\varepsilon_{j_{i}}^{2}\left(t\right)}{0.1\;\alpha_{j_{i}}^{2}}),\;0<\alpha_{j}\leq 1 \end{array}$$ (14)

which requires only one variable, *α*_*j*_, to describe the shape (as shown in Fig 3) but maintaining the same functionality. This also presents the advantage of simple application of a fuzzy inference engine, as described in a later section.

#### Task space adaptive control

*Task-space control* is designed in a similar manner to joint space. First, we define the error term in Cartesian space:

$$\begin{array}{ccc} e_{x}\left(t\right) & = & X\left(t\right)-X^{*}\left(t\right)\\ {\dot{e}}_{x}\left(t\right) & = & \dot{X}\left(t\right)-{\dot{X}}^{*}\left(t\right)\\ \varepsilon_{x}\left(t\right) & = & {\dot{e}}_{x}\left(t\right)+\kappa e_{x}\left(t\right) \end{array}$$ (15)

This leads to a change in the *feed-forward* and *feedback* terms described in Eq (12) to

$$\begin{array}{ccc} \delta F_{x} & \equiv & Q_{F}\;\varepsilon_{x}-\gamma_{x}\;F_{x}\\ \delta K_{x} & \equiv & Q_{K_{x}}\;\varepsilon_{x}e_{x}^{T}-\gamma_{x}\;K_{x}\\ \delta D_{x} & \equiv & Q_{D_{x}}\;\varepsilon_{x}{\dot{e}}_{x}^{T}-\gamma_{x}\;D_{x} \end{array}$$ (16)

so that

$$\begin{array}{c} \tau_{x}=J^{T}\left(q\right)\left(-F_{x}-K_{x}e_{x}-D_{x}{\dot{e}}_{x}\right) \end{array}$$ (17)

and the task-space forgetting factor is defined similarly to Eq (14), below:

$$\begin{array}{c} \gamma_{x}=\alpha_{x}\;\exp(-\frac{\varepsilon_{x}^{2}\left(t\right)}{0.1\;\alpha_{x}^{2}}),\;0<\alpha_{x}\leq 1\;. \end{array}$$ (18)

#### Hybrid Controller

The combination of the basic controller of Eq (7), the joint space controller of Eq (10) and the task space controller of Eq (17) yields the *hybrid controller*, and therefore the input torque *τ*_*u*_

$$\begin{array}{c} \tau_{u}\left(t\right)=\tau_{r}\left(t\right)+\tau_{x}\left(t\right)+\Omega \tau_{j}\left(t\right) \end{array}$$ (19)

where Ω ∈ ℜ^*n*×*n*^ is a weighting matrix, designed such that the joint torque feedback is limited to certain joints, dependent on the required task. Assuming an accurate dynamic model of the robot is available, the torques due to disturbance *τ*_*dist*_ are given as

$$\begin{array}{c} \tau_{dist}=M\left(q\right)\ddot{q}+C\left(q,\dot{q}\right)\dot{q}+G\left(q\right)-\tau_{u} \end{array}$$ (20)

i.e. the modeled system torques *minus* the input torque. By normalising this vector of torques to the maximum element, the weighting matrix Ω can be formed:

$$\begin{array}{c} \Omega_{ii}=\frac{\tau_{dist_{i}}}{\max_{1\leq i\leq n}\left(\tau_{dist}\right)}\;,\;\Omega_{ij}\equiv 0\;\left(i\neq j\right) \end{array}$$ (21)

which is then applied to Eq (19), so that joint-space control torque is applied primarily to those joints which are under the influence of large disturbance forces, and less to those which are not; this limits the control effort being applied unnecessarily, reducing the overall control effort that would otherwise be applied.

### Fuzzy Inference of Control Gains

Traditionally, the user sets the learning parameters *Q*_(⋅)_ and *α*_(⋅)_ based on experience of how the system responds at run-time, in order to ensure good control performance. Here, expert knowledge of the system is distilled into a fuzzy inference engine to tune the gains online, so that no prior user experience is required. An improvement in performance is also expected, as the system will pick appropriate gain values depending on the system response to unpredictable disturbances. Inferences are made according to the magnitudes of the tracking error and control effort, which we want to minimise, and also give a good indication of overall performance of the controller.

There are several steps required for fuzzy inference of an output *Y*. First, *fuzzification* maps a real scalar value (for example, temperature) into fuzzy space; this is achieved using *membership functions*. Let *X* be a space of points, with elements *x* ∈ *X* [31]. A fuzzy set *A* in *X* is described by a membership function *μ*_*A*_(*x*) associating a grade of membership *μ*_*A*_(*x*_*i*_) in the interval [0, 1] to each point *x* in *A*.

In this paper we use simple triangular membership functions, which have low sensitivity to change in input and are computationally inexpensive [32]. Additionally, from [32], all membership functions are set so that the *completeness* *ϵ* of all fuzzy sets is 0.5; this reduces uncertainty by eliminating areas in the universe of discourse with low degrees of truth, and also ensures reasonable overshoot, as described in [33].

Several definitions are required. A *union*, which corresponds to the connective OR, of two sets *A* and *B* is a fuzzy set *C*

$$\begin{array}{c} C=A\cup B;\;\mu_{C}\left(x\right)=\text{max}[(\mu_{A}\left(x\right),\mu_{B}\left(x\right)],\;x\in X \end{array}$$ (22)

An *intersection*, which corresponds to connective AND, can similarly be described:

$$\begin{array}{c} C=A\cap B;\;\mu_{C}\left(x\right)=\text{min}[(\mu_{A}\left(x\right),\mu_{B}\left(x\right)],\;x\in X \end{array}$$ (23)

The Cartesian product can be used to describe a relation between two or more fuzzy sets; let *A* be a set in universe *X* and *B* a set in universe *Y* [34]. The *Cartesian product* of *A* and *B* will result in a relation

$$\begin{array}{c} R\equiv A\times B\subset X\times Y \end{array}$$ (24)

where the fuzzy relation *R* has a membership function

$$\begin{array}{c} \mu_{R}\left(x,y\right)=\mu_{A\times B}\left(x,y\right)=\text{min}[\mu_{A}\left(x\right),\mu_{A}\left(y\right)] \end{array}$$ (25)

This is used in the Mamdani min-implication, to relate an input set to an output set, i.e. IF *x* is *A* THEN *y* is *B*. A rule set is then used to implicate the output, which is max-aggregated for all rules [25]. Defuzzification is then performed, using the common centroid method [35]. The defuzzified value y* is calculated using

$$\begin{array}{c} y^{*}=\frac{\int \mu_{B}\left(y\right)\;y\;dx}{\int \mu_{B}\left(y\right)\;dx} \end{array}$$ (26)

which computes the centre of mass of the aggregated output membership function, and relates the *μ* value back to a crisp output.

The raw inputs to our fuzzy systems are the joint-space tracking error and effort, *ɛ*_*j*_, *τ*_*u*_ and similarly, in task-space, *ɛ*_*x*_, *F*_*u*_. Before fuzzification can be performed, the inputs must be normalised so that the same inference engine is generic and is not dependent on the input magnitude. A baseline average of tracking errors ${\hat{ɛ}}_{j}\in {ℜ}^{n}$, ${\hat{ɛ}}_{x}\in {ℜ}^{6}$, input torque ${\hat{\tau }}_{u}\in {ℜ}^{n}$ and input force ${\hat{F}}_{u}\in {ℜ}^{6}$ are calculated for each degree of freedom over the total simulation time per time step $\frac{t_{f}}{\delta t}$:

$$\begin{array}{c} {\hat{\varepsilon }}_{x_{i}}=\frac{\sum |\varepsilon_{x_{i}}\left(t\right)|}{\frac{t_{f}}{\delta t}},\;{\hat{\varepsilon }}_{j_{i}}=\frac{\sum |\varepsilon_{j_{i}}\left(t\right)|}{\frac{t_{f}}{\delta t}},\;{\hat{\tau }}_{u_{i}}=\frac{\sum |\tau_{u_{i}}\left(t\right)|}{\frac{t_{f}}{\delta t}},\;{\hat{F}}_{u_{i}}=\frac{\sum |F_{u_{i}}\left(t\right)|}{\frac{t_{f}}{\delta t}}. \end{array}$$ (27)

These are then used to calculate the inputs to the fuzzy system, i.e. values which give an indication of performance compared to the previous iteration:

$$\begin{array}{c} {\bar{\varepsilon }}_{j_{i}}\left(t\right)=\frac{\sigma \;|\varepsilon_{j_{i}}\left(t\right)|}{{\hat{\varepsilon }}_{j_{i}}},\;{\bar{\varepsilon }}_{x_{i}}\left(t\right)=\frac{\sigma \;|\varepsilon_{x_{i}}\left(t\right)|}{{\hat{\varepsilon }}_{x_{i}}},\;{\bar{\tau }}_{u_{i}}\left(t\right)=\frac{\sigma \;|\tau_{u_{i}}\left(t\right)|}{{\hat{\tau }}_{u_{i}}},\;{\bar{F}}_{u_{i}}\left(t\right)=\frac{\sigma \;|F_{u_{i}}\left(t\right)|}{{\hat{F}}_{u_{i}}}. \end{array}$$ (28)

For all inputs to our fuzzy systems, a value less than *σ* indicates an improvement and values greater than *σ* indicate that performance is worse. Here we set *σ* = 0.5, so that the input range is roughly between 0 and 1. There is no upper limit to the variables generated in Eq (28), so any input above unity returns a maximum truth value in the ‘high’ classification. This allows a generic set of input membership functions to be applied to all systems.

These normalised variables are then used in the adaptive laws Eqs (12), (14), (16) and (18) as $Q_{\tau }\equiv Q_{\tau }({\bar{ɛ}}_{j},{\bar{\tau }}_{j})$, $Q_{Kj}\equiv Q_{Kj}({\bar{ɛ}}_{j},{\bar{\tau }}_{j})$, $Q_{Dj}\equiv Q_{Dj}({\bar{ɛ}}_{j},{\bar{\tau }}_{j})$, $\alpha_{j}\equiv \alpha_{j}({\bar{ɛ}}_{j},{\bar{\tau }}_{j})$ for the joint-space controller, and correspondingly for the task-space controller.

The rules for fuzzy inference of the control gains are set using expert knowledge. In general: IF control effort is too high THEN gain is set low; IF tracking error is poor THEN gain is set high, as shown in Table 2 for *Q*_(⋅)_. The truth table for the forgetting factor gain (Table 3) is slightly different, in that *α* is required to be *larger* when tracking error is improved. Note that *Q*_(⋅)_ and *α*, the outputs of the fuzzy inference system, are bounded:

$$\begin{array}{c} 0<Q_{(\cdot )ii}\leq Q_{(\cdot )ii}max\\ 0<\alpha_{i}\leq \alpha_{i}max \end{array}$$ (29)

where the maximum values are set according to previous trials performed without application of the fuzzy system.

**Table 2:** Truth table for inference of output *Q*_(⋅)_ based on fuzzy memberships of ${\bar{ɛ}}_{j_{i}},{\bar{ɛ}}_{x_{i}},{\bar{\tau }}_{u_{i}}$, ${\bar{F}}_{u_{i}}$.

**Input**					**Output**	
*R*_1_:	**IF**	${\bar{ɛ}}_{j},{\bar{ɛ}}_{x}<\sigma$			**THEN**	*Q*_(⋅)_ *low*
*R*_2_:	**IF**	${\bar{ɛ}}_{j},{\bar{ɛ}}_{x}\approx \sigma$	and	${\bar{\tau }}_{u},{\bar{F}}_{u}<\sigma$	**THEN**	*Q*_(⋅)_ *low*
*R*_3_:	**IF**		and	${\bar{\tau }}_{u},{\bar{F}}_{u}\geq \sigma$	**THEN**	*Q*_(⋅)_ *medium*
*R*_4_:	**IF**	${\bar{ɛ}}_{j},{\bar{ɛ}}_{x}>\sigma$	and	${\bar{\tau }}_{u},{\bar{F}}_{u}\leq \sigma$	**THEN**	*Q*_(⋅)_ *high*
*R*_5_:	**IF**		and	${\bar{\tau }}_{u},{\bar{F}}_{u}>\sigma$	**THEN**	*Q*_(⋅)_ *medium*

**Table 3:** Truth tables for inference of output *α*_(⋅)_ based on fuzzy memberships of ${\bar{ɛ}}_{j_{i}},{\bar{ɛ}}_{x_{i}},{\bar{\tau }}_{u_{i}}$, ${\bar{F}}_{u_{i}}$.

**Input**					**Output**	
*R*_1_:	**IF**	${\bar{ɛ}}_{j},{\bar{ɛ}}_{x}<\sigma$	and	${\bar{\tau }}_{u},{\bar{F}}_{u}<\sigma$	**THEN**	*α* *medium*
*R*_2_:	**IF**		and	${\bar{\tau }}_{u},{\bar{F}}_{u}\geq \sigma$	**THEN**	*α* *high*
*R*_3_:	**IF**	${\bar{ɛ}}_{j},{\bar{ɛ}}_{x}\approx \sigma$	and	${\bar{\tau }}_{u},{\bar{F}}_{u}\leq \sigma$	**THEN**	*α* *medium*
*R*_4_:	**IF**		and	${\bar{\tau }}_{u},{\bar{F}}_{u}>\sigma$	**THEN**	*α* *high*
*R*_5_:	**IF**	${\bar{ɛ}}_{j},{\bar{ɛ}}_{x}>\sigma$	and	${\bar{\tau }}_{u},{\bar{F}}_{u}\leq \sigma$	**THEN**	*α* *low*
*R*_6_:	**IF**		and	${\bar{\tau }}_{u},{\bar{F}}_{u}>\sigma$	**THEN**	*α* *medium*

**Fig 4:**
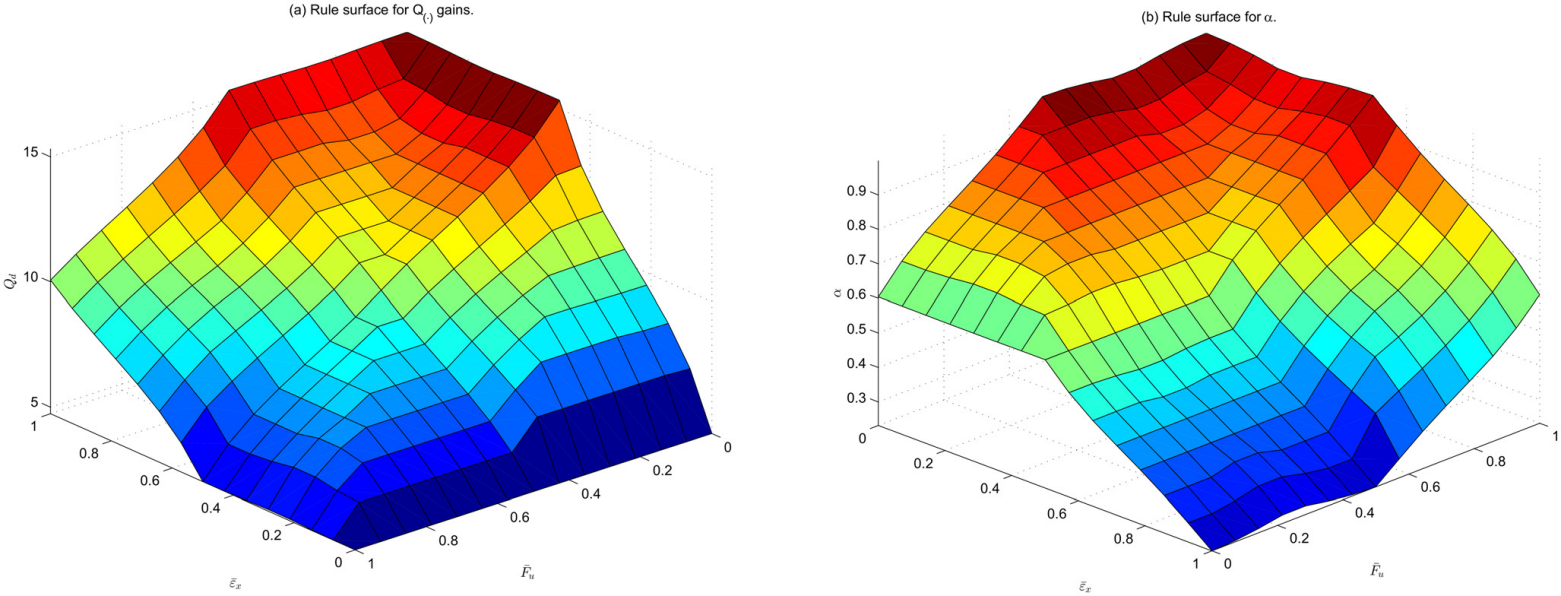
Surface plots showing rule surfaces. (a): adaptation gain *Q*_*Dx*_, and (b): value of *α*_*x*_, based on inputs ${\bar{ɛ}}_{x}$ and ${\bar{F}}_{u}$ described in Eq (28). Task-space gains are characterised by a similar surface.

How changes in control effort and tracking error affect the *Q*_(⋅)_ gains is shown in Fig 4(a). It can be seen that in general: gain increases when tracking error is high and control effort is low, and minimal gain occurs when tracking error is low and control effort is high. The surface of fuzzy inference of *α* is shown in Fig 4(b) where it can be seen that the forgetting factor will be at its greatest when tracking error is low and control effort is high.

### Stability

The stability of the controller in joint space and convergence to a small bounded set were shown in [9], and the proof for the Cartesian space controller is similar. However, here the diagonal adaptation gain matrices *Q*_(⋅)_ are time varying, which must be taken into account. From [9] Appendix C, the difference in energy of the system *δV*(*k*) = *δV*_*p*_(*t*)+*δV*_*c*_(*t*) is shown to converge to zero. No change to the derivation of the first part *δV*_*p*_(*t*) is needed here, so that section of the proof still holds. A change is made in comparison to [9], equations (39–41) where $Q_{\left(\cdot \right)}^{-1}$ is replaced with $Q_{\left(\cdot \right)}^{-1}(\sigma )$ so that

$$\begin{array}{ccc} \delta V_{c}\left(t\right)=\frac{1}{2}\int_{t-\delta t}^{t} & \{ & \text{tr}({\tilde{K}}^{T}\left(\sigma \right)Q_{K}^{-1}\left(\sigma \right)\tilde{K}\left(\sigma \right)-{\tilde{K}}^{T}\left(\sigma -\delta t\right)Q_{K}^{-1}\left(\sigma -\delta t\right)\tilde{K}\left(\sigma -\delta t\right))\\ & + & \;\text{tr}({\tilde{D}}^{T}\left(\sigma \right)Q_{D}^{-1}\left(\sigma \right)\tilde{D}\left(\sigma \right)-{\tilde{D}}^{T}\left(\sigma -\delta t\right)Q_{D}^{-1}\left(\sigma -\delta t\right)\tilde{D}\left(\sigma -\delta t\right))\\ & + & \;{\tilde{\tau }}^{T}\left(\sigma \right)Q_{\tau }^{-1}\left(\sigma \right)\tilde{\tau }\left(\sigma \right)-{\tilde{\tau }}^{T}\left(\sigma -\delta t\right)Q_{\tau }^{-1}\left(\sigma -\delta t\right)\tilde{\tau }\left(\sigma -\delta t\right)\}. \end{array}$$ (30)

Defining a new variable *δQ* ≡ diag[I ⊗ *δQ*_*K*_, I ⊗ *δQ*_*D*_, I ⊗ *δQ*_*τ*_] (where ⊗ is the Kronecker product) allows us to add another term to the end of [9](44), producing

$$\begin{array}{ccc} \delta V_{c}\left(t\right) & = & -\frac{1}{2}\int_{t-\delta t}^{t}\delta {\tilde{\Phi }}^{T}\left(\sigma \right)Q^{-1}\left(\sigma \right)\delta \tilde{\Phi }\left(\sigma \right)\;d\sigma -\int_{t-\delta t}^{t}\gamma \left(\sigma \right)Q^{-1}\left(\sigma \right){\tilde{\Phi }}^{T}\left(\sigma \right)\Phi \left(\sigma \right)\;d\sigma \\ & & +\;\int_{t-\delta t}^{t}\varepsilon^{T}\left(\sigma \right)\tilde{K}\left(\sigma \right)e\left(\sigma \right)+\varepsilon^{T}\left(\sigma \right)\tilde{D}\left(\sigma \right)\dot{e}\left(\sigma \right)+\varepsilon^{T}\left(\sigma \right)\tilde{\tau }\left(\sigma \right)\;d\sigma \\ & & +\;\int_{t-\delta t}^{t}{\tilde{\Phi }}^{T}\left(\sigma \right)\delta Q^{-1}\left(\sigma \right)\tilde{\Phi }\left(\sigma \right)\;d\sigma . \end{array}$$ (31)

The term inside the last integrand can be described by ${ɛ}_{Q}{\tilde{\Phi }}^{T}\tilde{\Phi }$ where

$$\begin{array}{c} \varepsilon_{Q}{\tilde{\Phi }}^{T}\tilde{\Phi }\geq \text{tr}(\varepsilon_{K}{\tilde{K}}^{T}\tilde{K}+\varepsilon_{D}{\tilde{D}}^{T}\tilde{D}+\varepsilon_{\tau }{\tilde{\tau }}^{T}\tilde{\tau }),\;\varepsilon_{Q}=\max\left(\varepsilon_{K},\varepsilon_{D},\varepsilon_{\tau }\right) \end{array}$$ (32)

given that ${\tilde{K}}^{T}\Phi_{K}^{-1}\tilde{K}\leq {ɛ}_{K}{\tilde{K}}^{T}\tilde{K}$, ${\tilde{D}}^{T}\Phi_{D}^{-1}\tilde{D}\leq {ɛ}_{D}{\tilde{D}}^{T}\tilde{D}$ and ${\tilde{\tau }}^{T}\Phi_{\tau }^{-1}\tilde{\tau }\leq {ɛ}_{\tau }{\tilde{\tau }}^{T}\tilde{\tau }$, where *ɛ*_*K*,*D*,*τ*_ are defined as the minimum eigenvalues of $\Phi_{K,D,\tau }^{-1}$. This can then be added to the condition in [9](46) which gives the inequality

$$\begin{array}{c} \delta V\;\geq \;\lambda_{L}{∥\varepsilon ∥}^{2}+{\bar{\gamma }}_{max}{∥\tilde{\Phi }∥}^{2}-\gamma^{\prime }∥\tilde{\Phi }∥∥\Phi^{*}∥\;\geq \;\lambda_{L}{∥\varepsilon ∥}^{2}+\bar{\gamma }{∥\tilde{\Phi }∥}^{2}-\gamma^{\prime }∥\tilde{\Phi }∥∥\Phi^{*}∥\;\geq \;0 \end{array}$$ (33)

where *γ*′ = *Q* ^−1^ *γ*, and $\bar{\gamma }=\gamma^{\prime }+{ɛ}_{Q}$. This is a sufficient condition to prove stability, following the details in appendix C of [9], and given that *Q*(*t*) is bounded by the output of fuzzy inference stipulated in Eq (29).

## Simulations

The task consisted of tracking a smooth minimal jerk trajectory along the *y* coordinate defined as:

$$\begin{array}{c} y^{*}\left(t\right)\equiv y^{*}\left(0\right)+(y^{*}\left(T\right)-y^{*}\left(0\right))\left(10{\bar{t}}^{3}-15{\bar{t}}^{4}+6{\bar{t}}^{5}\right),\;\bar{t}\equiv \frac{2t}{T} \end{array}$$ (34)

where *T* is the movement duration. Joint-space angular velocity is computed using the pseudo inverse *J* ^†^(*q*) ≡ *J* ^*T*^(*JJ* ^*T*^)^−1^ of the Jacobian, through

$$\begin{array}{c} {\dot{q}}^{*}\left(t\right)=J^{†}\left(q\right)\;{\left[0,y^{*}\left(t\right),0,0,0,0\right]}^{T}, \end{array}$$ (35)

from which the position and acceleration can be found respectively using

$$\begin{array}{c} q^{*}\left(t\right)\equiv \int_{0}^{t}{\dot{q}}^{*}\left(t\right)\;dt\;,\;{\ddot{q}}^{*}\equiv \frac{d}{dt}({\dot{q}}^{*}\left(t\right)). \end{array}$$ (36)

Simulations of the proposed task and controller were performed using MATLAB with a kinematic and dynamic Baxter robot rigid joint model, implemented using Peter Corke’s Robotics Toolbox [36, 37]. To test the controller under continuous different conditions, the two disturbance forces *F*_*envt*_ and *F*_*task*_ were introduced in different phases:

- Phase I: No disturbance;
- Phase II: *F*_*task*_ only;
- Phase III: *F*_*envt*_ only;
- Phase IV: *F*_*envt*_ and *F*_*task*_.

Performance was analysed in each phase, to observe the controller’s reaction to different perturbations. It was expected that joint-space control would improve rejection of *F*_*envt*_, and task-space control to reject disturbance caused by *F*_*task*_; the order of phases was set so that the adaptation progress would be easier for readers to understand. A performance index, *η*, was calculated from the integral of the product of input force *F*_*u*_ and task-space tracking error *ɛ*_*x*_:

$$\begin{array}{c} \eta =\int_{t_{s}}^{t_{f}}F_{u}{\left(t\right)}^{T}QF_{u}\left(t\right)+\varepsilon_{x}^{T}\left(t\right)R\;\varepsilon_{x}\left(t\right)\;dt \end{array}$$ (37)

where *Q*, *R* ∈ ℜ^6×6^ are positive diagonal scaling matrices, and *t*_*s*_ and *t*_*f*_ were set to obtain *η* for each phase of the simulation. A small performance index *η* corresponds to small tracking error and control effort, and thus indicates good performance.

## Results

### Hybrid Control

Performance of the hybrid controller *τ*_*u*_(*t*) = *τ*_*r*_(*t*)+*τ*_*x*_(*t*)+Ω*τ*_*j*_(*t*) was compared against the controller in joint-space only, when *τ*_*u*_(*t*) = *τ*_*r*_(*t*)+*τ*_*j*_(*t*), and in task-space only, where *τ*_*u*_(*t*) = *τ*_*r*_(*t*)+*τ*_*x*_(*t*). Disturbance parameters remain the same in each case; for *F*_*task*_(*t*) defined in Eq (2), *p* = 20 sin(2*π* 50 *t*), and for *F*_*envt*_(*t*) from Eq (4) the parameters are *r* = 100 sin(2*π* 0.1042 *t*). The trajectory period and travel distance were set to 4.8s and 0.2m respectively. Each simulation phase corresponds to one completion of the trajectory of Eq (34).

**Fig 5:**
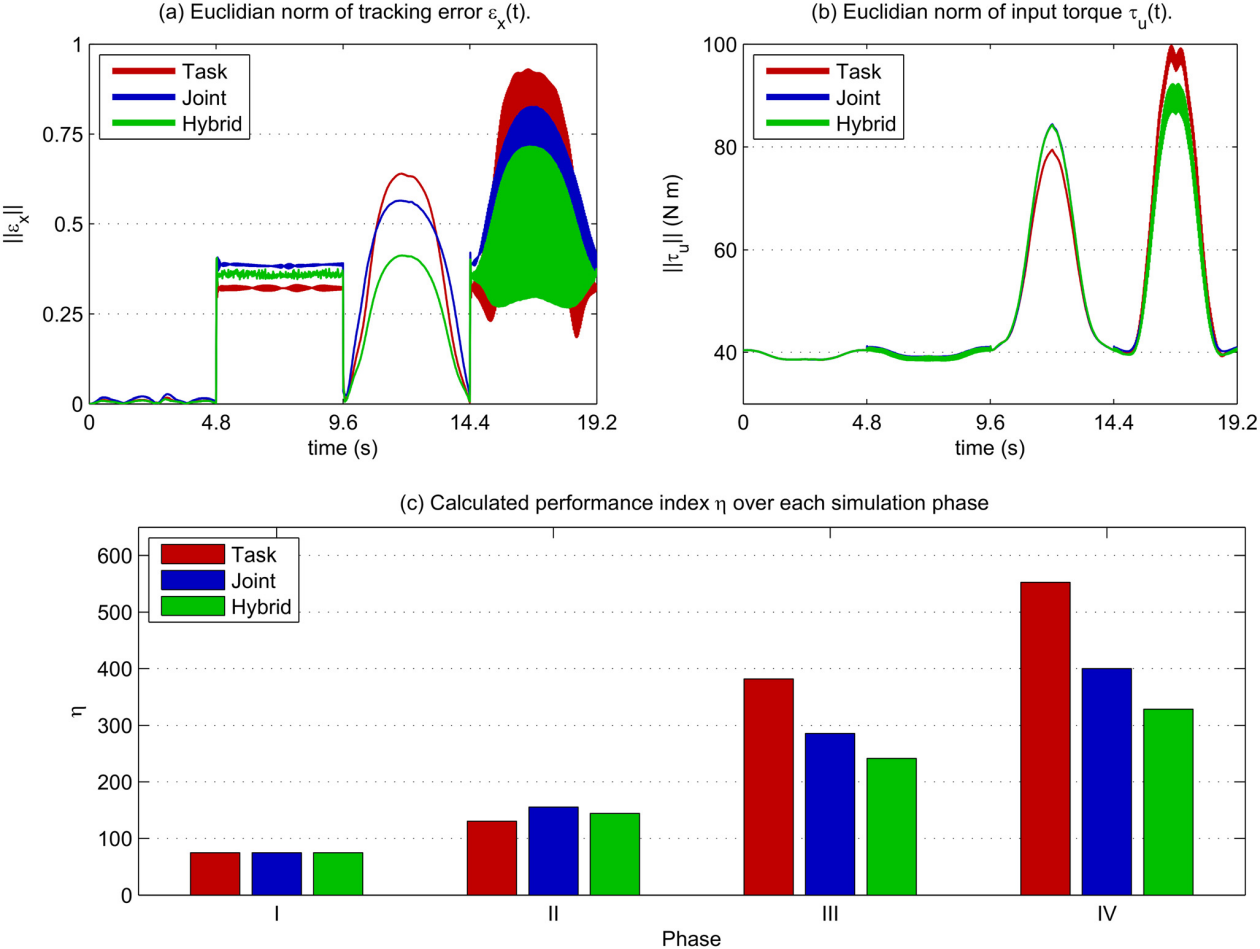
Comparison of controllers performance. (a): In the first phase (0 < *t* < 4.8) little difference can be observed in tracking error for the three controllers. In phase II task-space has the lowest error, and joint space the highest, with the hybrid control in between, as expected due to the disturbance type. In the next two phases (9.6 < *t* < 19.2) task-space control produces the highest error, while the hybrid controller shows a much lower tracking error than its component parts. (b): Examining the input torques *τ*_*u*_ little difference can be seen between the three control schemes. (c): The performance index *η* in each phase demonstrates the limitations of each control type under different disturbance conditions. In particular task-space control performance is degraded in phases III, IV where joint-space is superior. Hybrid control shows improved performance over both.

The Cartesian tracking error *ɛ*_*x*_ in Fig 5(a) for all three control schemes shows how task-space performs better when a tool-type disturbance is applied, but suffers when a large disturbance is applied away from the end-effector. In this case, joint-space control was able to more effectively reduce tracking error. When combined in the hybrid controller, tracking error was reduced further. From Fig 5(b) it can be noted that there was little difference in the overall amount of control effort being applied between the three methods. The measures of tracking error and control effort were combined to form the performance index *η* for each phase, shown in Fig 5(c). A clear difference could be seen in the performances of the task-space and joint-space controllers between phases II and III, where the disturbance type was switched from *F*_*task*_ to *F*_*envt*_; task-space control was better at handling the former, and joint-space the latter. The hybrid controller showed a slight improvement over joint-space in phase II but exhibited an improvement over its component parts in phases III and IV. Considering ||*τ*_*u*_|| was similar for all three, as seen in Fig 5(b), this suggests that the hybrid control was applying control in a more targeted fashion, i.e. only applying additional feedback to the joints which require it.

**Fig 6:**
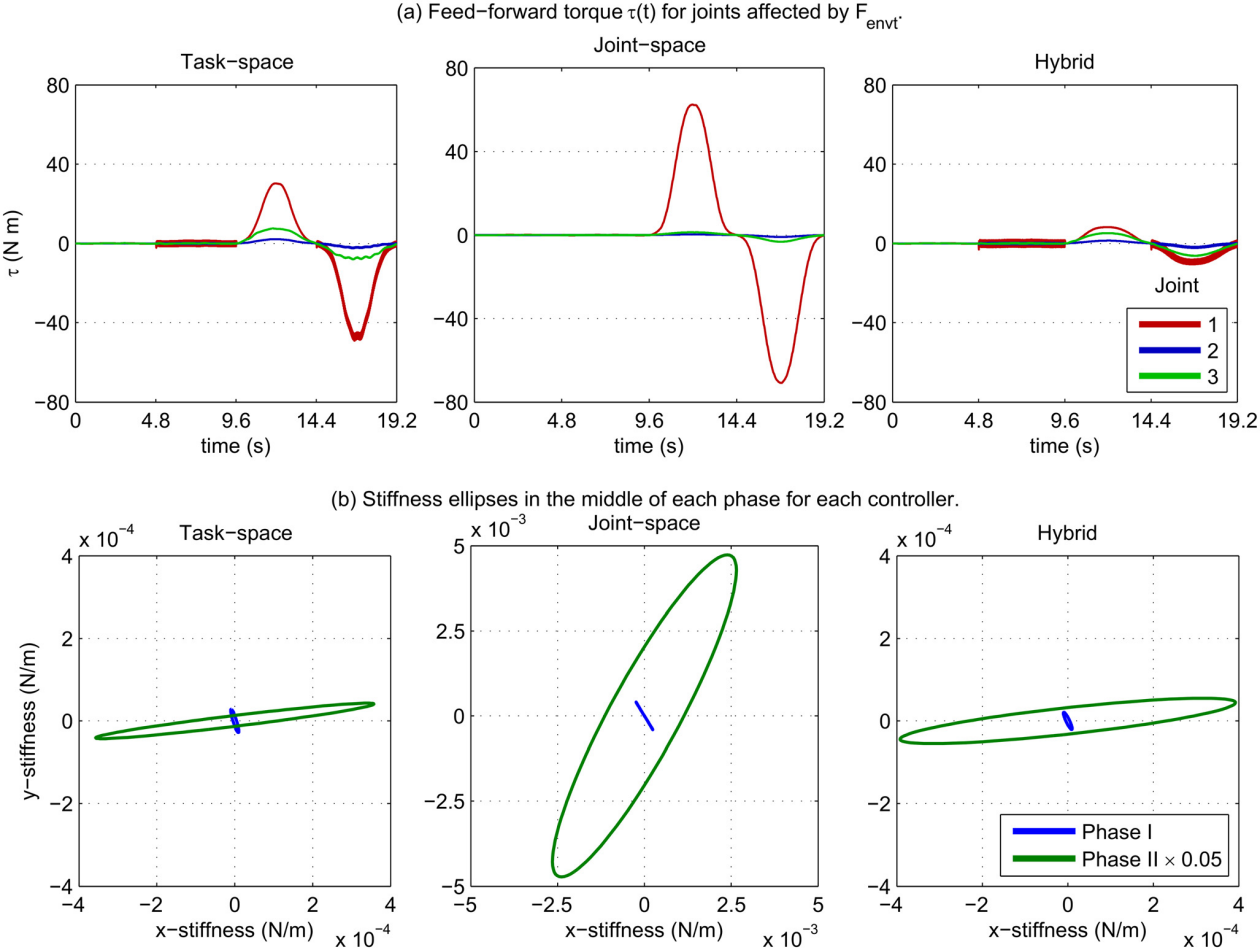
Learned feed-forward torque and stiffness. In (a) we can see how the feedforward torque increases in the last two phases to compensate for the low frequency disturbance. (b) Comparison of stiffness geometry represented by ellipses in the *x* and *y* planes, of midpoint of phases I—II, for each controller. Note for task-space and hybrid control the ellipse is elongated primarily in the *x*-axis corresponding to the perturbation direction.

By examining the evolution of feed-forward torque in Fig 6(a) we see how in phases III and IV large increases were made to compensate for the low frequency *F*_*envt*_ disturbance, predominantly in the first joint (the rotation of which is aligned with the x-y plane). Comparing the magnitude of feed-forward torque between controllers it is clear that joint-space control generated much higher torques, while hybrid control torques were much lower and less weighted towards joint 1.

Cartesian stiffness ellipses are shown in Fig 6(b); In task-space and hybrid control, it can be observed how the stiffness changed from a slight orientation in the *y*-direction (due to the trajectory moving along this axis) to a much larger ellipse predominantly in the *x*-axis: aligned with the direction of disturbance. Joint-space control, however, produced ellipses less-aligned with the direction of disturbance. This shows that feedback torque is being applied inefficiently in this case.

### Fuzzy Inference of Control Gains

The effectiveness of the fuzzy inference of control gains *Q*_(⋅)_ and *α* was tested through implementation on the hybrid controller, and compared against results obtained in the previous section (where control gains are fixed). Base-line averages described in Eq (28) and upper limits of adaptation gains were calculated from data collected running the hybrid controller in the previous experiment, which were then used as the input to the fuzzy engines affecting the adaptive laws.

**Fig 7:**
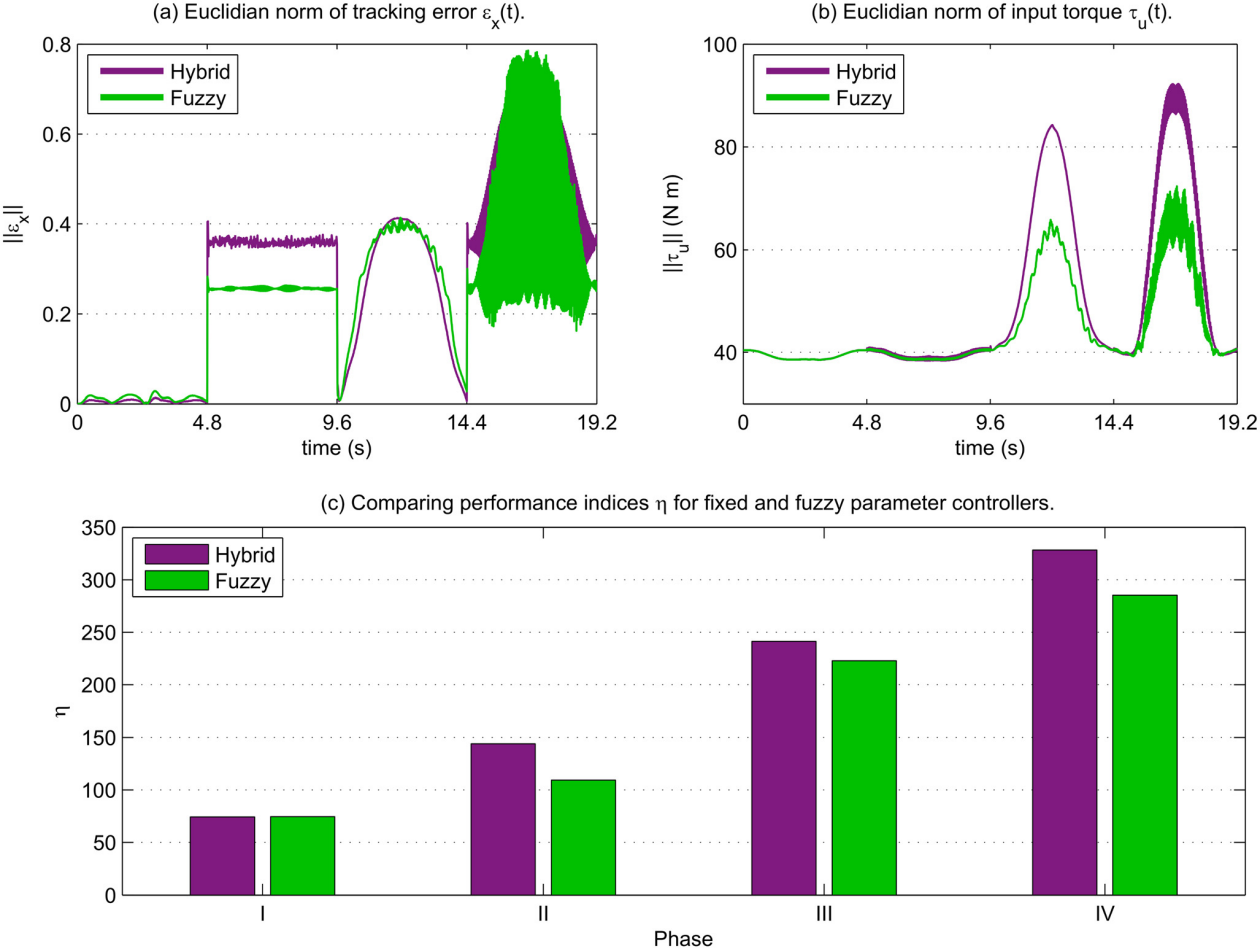
Performance from fuzzy tuning of learning parameters. In (a), tracking error for the hybrid controller (green) is compared to the same controller with fuzzy tuning of adaptive parameters (purple). (b): Comparison of input control torques for the two control schemes. (c): Performance indices calculated for each phase, showing an improvement for all phases where disturbance is present.

By examining Fig 7(a) we can see that there was an improvement in tracking error in phase II, but not so much in other phases, where it is similar to previous results. However, by comparing the results with Fig 7(b) we can see that although control torque was not reduced in the first two phases, there was a significant reduction in the last two; this demonstrates not only that the online tuning is able to reduce tracking error when control effort is already minimal, but also reduces the control effort required to maintain good tracking. This is reflected in Fig 7(c) which shows in all disturbance phases that the aggregate performance index score was improved by tuning the learning parameters online.

**Fig 8:**
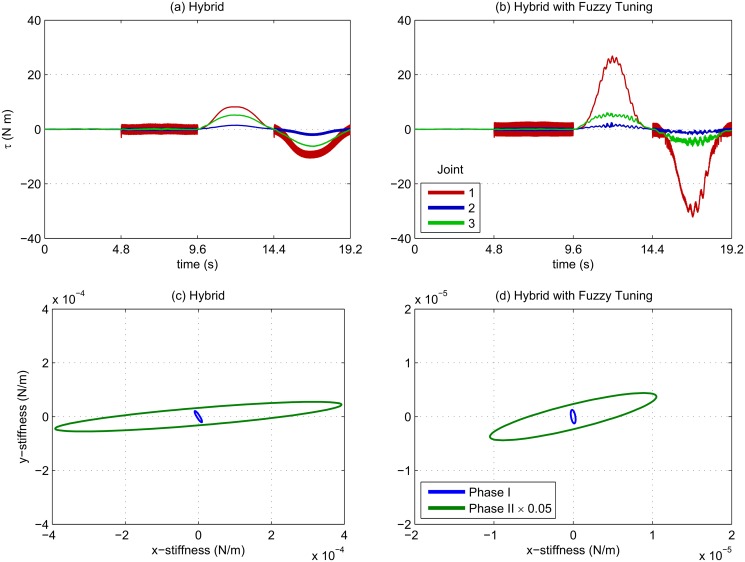
Force and impedance with and without fuzzy inference. (a), (b): The shape of evolution through time is similar between the two controllers; however, the fuzzy hybrid controller applies a larger feed-forward torque. (c), (d): Ellipses are for the hybrid controller have a higher magnitude than the same controller with fuzzy parameter tuning; note that scaling in the fuzzy tuning case is ×0.02 scaled. Ellipses in the second phase are elongated in the direction of disturbance.

In Fig 8(a) and 8(b) the feed-forward torques of the proximal joints are compared. We can see that the fuzzy tuning had a much higher response amplitude, although the shape has remained the same. Compared with Fig 8(c) and 8(d) the stiffness ellipse displays a reduced magnitude with fuzzy tuning. This suggests that the online-tuned controller increased feed-forward torque while sacrificing stiffness to reduce the control effort observed in Fig 5(b), although the geometry of the ellipse was maintained in the direction of disturbance.

## Conclusions

This paper investigated the ideas of combining joint-space and task-space feedback control to create a hybrid controller, and of online fuzzy tuning of learning parameters.

The controller was based on a bio-inspired design, which has been shown to acquire stable and successful performance with minimal effort. The controller was implemented on a dynamic model of the redundant Baxter robot arm. The results show that the hybrid controller displays reductions in tracking error of around 26% and 16% on average for the task and joint-space controllers respectively, with only a 6% maximum increase in control effort. Thus, demonstrating the hybrid controller is able to benefit from both joint-space and Cartesian-based control, providing robustness against disturbances occurring at the end-effector or any point along the arm.

The results further show how fuzzy inference can be used to set the learning parameters automatically, instead of the normal practice of setting them manually. The simulation results demonstrate an average 24% reduction in control effort and 15% improvement in overall performance with this fuzzy meta-learning than with fixed learning parameters, as well as avoiding the need for trial testing to select optimum values for adaptation gains. We also note that the method used to normalise inputs to the fuzzy system may enable iterative performance improvement, as the performance of the current iteration is compared against the previous, and the fuzzy system seeks to reduce tracking error and control effort as much as possible.
